# Editorial: Biomarkers for stroke recovery

**DOI:** 10.3389/fneur.2023.1170308

**Published:** 2023-03-07

**Authors:** Pradeep Kumar, Keith Pennypacker

**Affiliations:** ^1^Clinical Research Unit, All India Institute of Medical Sciences, New Delhi, India; ^2^Department of Neurology and Neuroscience, University of Kentucky, Lexington, KY, United States

**Keywords:** stroke, biomarkers, recovery, ischemic stroke, hemorrhagic stroke

Stroke is a significant contributor to disability and mortality worldwide, posing a substantial burden on individuals, families, and healthcare systems (1, 2). Despite advancements in acute interventions like thrombolysis and mechanical thrombectomy, stroke patients' post-stroke recovery process remains intricate and multi-dimensional, often falling short of optimal patient outcomes (3–5). Several factors influence the complex nature of stroke recovery, such as stroke severity, lesion location, comorbidities, and patient characteristics (6–8). To improve stroke care, it is crucial to comprehend the biological mechanisms underlying stroke recovery and identify reliable biomarkers that can predict recovery outcomes (9–11).

Blood biomarkers are advantageous as a source of biomarkers due to their easy accessibility, non-invasiveness, and the ability to detect systemic changes associated with stroke (12, 13). Several promising blood biomarkers, such as inflammatory markers like C-reactive protein (CRP) and interleukin-6 (IL-6), as well as neurotrophic factors like brain-derived neurotrophic factor (BDNF) and vascular endothelial growth factor (VEGF), have been associated with stroke recovery (14–17). While these biomarkers show potential as prognostic tools, their reliability is limited due to patient variability resulting from co-morbidities, demographics, and the type of stroke (18, 19).

The Research Topic titled “*Biomarkers for stroke recovery*” in Frontiers in Neurology was aimed at providing new insights into the application of biomarkers in predicting stroke outcomes and identifying potential treatment targets. It comprises 19 articles that investigate different biomarkers for stroke recovery. Zhou et al. found that neurofilament light chain and S100B serum levels were associated with disease severity and outcome in patients with aneurysmal subarachnoid hemorrhage (aSAH). This study highlights the potential of these biomarkers in predicting outcomes and monitoring disease progression in aSAH patients. He et al. investigated the relationship between hyper-homocysteinemia and poor postoperative angiogenesis in adult patients with Moyamoya disease. The study found that hyperhomocysteinemia was a predictor for poor postoperative angiogenesis with this particular cerebrovascular disease. Using a discovery-based SWATH-MS proteomic approach, Misra et al. identified a panel of blood-based protein biomarkers for the diagnosis of acute stroke. The study highlights the potential for these biomarkers to enable earlier treatment and improve patient outcomes. Ye et al. developed and validated a nomogram that utilizes serum amyloid A as a biomarker to predict the risk of cognitive impairment after lacunar infarction. The authors suggest that this blood-based biomarker has potential for identifying at-risk patients and facilitating early interventions to improve patient outcomes treatment strategies. Wang et al. conducted a matched cohort study to determine the relationship between the mean of 24-h venous blood glucose and in-hospital mortality among patients with subarachnoid hemorrhage. The study found that high mean 24-h venous blood glucose levels were associated with increased in-hospital mortality, which could provide an early warning for additional care for these patients.

Mo et al. explored the dual function of microglial polarization and its treatment targets in ischemic stroke. The study found that regulating microglial polarization could be a potential treatment strategy for ischemic stroke. Zhong C. et al. analyzed the association between high serum anion gap and all-cause mortality in non-traumatic subarachnoid hemorrhage. The study found that a high serum anion gap was correlated with increased all-cause mortality in patients with non-traumatic subarachnoid hemorrhage. Peng et al. investigated the relationship between red blood cell distribution width and post-stroke fatigue in the acute phase of acute ischemic stroke. High red blood cell distribution width levels were associated with increased post-stroke fatigue that affects many stroke patients.

Chen et al. evaluated the predictive value of the neutrophil-to-lymphocyte ratio on stroke outcome after intravenous thrombolysis and mechanical thrombectomy. High neutrophil-to-lymphocyte ratio on day 1 was a predictor of poor stroke outcome, which could be used for early identification of patients requiring additional care. Zhong J. et al. investigated the predictive value of reduced plasma levels of RGM-A on stroke-associated pneumonia in patients with acute ischemic stroke, in which stroke patient have a 30% infection rate leading to poorer clinical outcomes (20). The study found that reduced plasma levels of RGM-A could predict stroke-associated pneumonia in patients with acute ischemic stroke. Zhang et al. evaluated the association of the systemic immune-inflammation index (SII) with carotid plaque vulnerability in patients.

Chen et al. investigated the longitudinal changes in the hypothalamic-pituitary-adrenal axis and sympathetic nervous system in relation to stroke prognosis. The authors demonstrated that changes in these systems are linked to worse patient outcomes, highlighting the need for targeted interventions to improve patient outcomes. Yao et al. examined the effect of mean heart rate on 30-day mortality in ischemic stroke patients with atrial fibrillation using data from the MIMIC-IV database. Their findings suggest that higher mean heart rate is associated with an increased risk of mortality, underscoring the need for targeted interventions to reduce heart rate and improve patient outcomes. A systematic review and meta-analysis by Huang et al. investigated the association of stress hyperglycemia ratio (SHR) with clinical outcomes in patients with stroke. The study found that elevated SHR was significantly associated with poor outcomes, including increased mortality, disability, and length of hospital stay. This suggests that monitoring SHR may be a useful biomarker for predicting stroke recovery. Another study by Jiang et al. explored the use of the monocyte-to-lymphocyte ratio (MLR) as a biomarker for acute kidney injury (AKI) after acute hemorrhagic stroke. The results indicated that MLR was a significant predictor of AKI, and higher MLR values were associated with a greater risk of AKI. This finding highlights the potential of MLR as a prognostic biomarker for stroke recovery. In addition, Guo et al. investigated the use of optic nerve sheath diameter (ONSD) and ONSD/eyeball transverse diameter (ETD) ratio as biomarkers for predicting malignant progression in ischemic stroke. The study found that elevated ONSD and ONSD/ETD ratio were significantly associated with malignant progression, indicating their potential as biomarkers for stroke recovery.

Another promising avenue of biomarker research in stroke recovery is the use of imaging biomarkers. Neuroimaging techniques, such as magnetic resonance imaging (MRI) and positron emission tomography (PET), can provide insights into structural and functional changes in the brain following stroke. Several imaging biomarkers have been proposed as potential predictors of recovery outcomes, including measures of white matter integrity, cortical thickness, and functional connectivity. Imaging biomarkers offer unique advantages, such as the ability to detect changes in specific brain regions, their utility is limited by the high cost and technical expertise required for acquisition and analysis. Wei et al. investigated the potential of combining cerebral small vessel disease with cerebral collaterals to predict the prognosis of patients with acute large artery atherosclerotic stroke. Their study suggests that this approach could be useful for predicting patient outcomes and developing personalized treatment strategies. Tahmi et al. evaluated neuroimaging biomarkers of cognitive recovery after ischemic stroke. The study found that different neuroimaging techniques could be used to predict cognitive recovery after ischemic stroke. In addition, there is a need for larger, multicentric studies that can validate and replicate findings across different populations and settings.

In conclusion, the “*Biomarkers for stroke recovery*” Research Topic provides a comprehensive overview of the latest advances in this field. The 19 studies included in this topic demonstrate the potential of biomarkers to predict stroke recovery and improve treatment outcomes. The research presented in this topic underscores the importance of further investigations to validate these biomarkers and their role in clinical practice. We hope that this Research Topic will stimulate further research in this field and pave the way for improved stroke recovery outcomes.

## Author contributions

PK conceptualized the idea of this research topic and drafted the editorial. KP contributed to writing the editorial to its final version. Both authors approved the submitted version for publication.
